# Analyzing the Correlation Between Surgeon Experience and Patient Length of Hospital Stay

**DOI:** 10.7759/cureus.10099

**Published:** 2020-08-28

**Authors:** Sharad Rajpal, Mancy Shah, Niketna Vivek, Sigita Burneikiene

**Affiliations:** 1 Neurosurgery, Boulder Neurosurgical and Spine Associates, Boulder, USA; 2 Medicine, University of Colorado Boulder, Boulder, USA; 3 Neurosurgery, Justin Parker Neurological Institute, Boulder, USA

**Keywords:** length of hospital stay, spinal fusion, surgeon experience

## Abstract

Introduction

Many clinical, social, and even economic factors have been extensively analyzed in the literature and shown to influence the length of stay (LOS) after spinal procedures. However, surgeon’s experience was mostly examined relative to a learning curve and not regarding the time in practice. The primary objective of this study was to determine the effect of one surgeon’s experience on the LOS in patients undergoing one- to two-level transforaminal lumbar interbody fusions (TLIFs).

Materials and Methods

The study design was a retrospective cohort study of hospital discharge data. The cohort was comprised of 240 consecutive patients who had undergone open one- or two-level elective TLIF procedures for lumbar degenerative disc disease. The primary predictor was the surgeon’s experience based upon the years of practice. The primary outcome was LOS, which was controlled by the discharge criteria that remained consistent throughout the study.

Results

Based on the Poisson regression model, it can be inferred that the LOS is not significantly associated with a surgeon’s experience (Pr(>|t|) = 0.8985, CI: -0.5825 to 0.5114) while controlling for all other variables. Other independent factors did seem to significantly influence patients’ LOS, including the admission type (Pr(>|t|) = 9.637^-08^, CI: -0.8186 to -0.3786), the number of TLIF levels (Pr(>|t|) = 1.721^-06^, CI: 0.0606 to 0.1446), the Clavien-Dindo ( Pr(>|t|) = 0, CI: 0.1489 to 0.1494), the American Society of Anesthesiologists (ASA) physical status classification scores (Pr(>|t|) = 4.878^-3^, CI: 0.0336 to 0.1880), and being discharged to skilled nursing facility (Pr(>|t|) = 3.44^-2^, CI: 0.0127 to 0.3339).

Conclusions

Based upon the years in practice, surgeon experience was not associated with length of hospitalization and estimated blood loss during surgery in patients undergoing one- and two-level TLIF surgeries. However, while controlling for all other variables, the surgeon’s experience and surgical time had a highly significant correlation. The study results clearly demonstrated efficiency, but we did not identify a clear correlation between LOS and surgeon experience overtime suggesting that other factors are likely contributing to such outcome. The average LOS is a complex measure of healthcare resource use and hospital discharge policy or other variables are likely having more effect on LOS than individual surgeons’ preferences.

## Introduction

The acquisition and development of skills are critical components of any surgeon’s training, but the volume of cases and years in practice are the two main factors that determine surgical performance outcomes [1,2]. It has been demonstrated that increased surgical volume was associated with lower readmission rates [3] and perioperative complications [3,4] in patients undergoing spinal surgeries. Surgeon’s experience has also been linked to surgery duration and blood loss [5,6], the accuracy of pedicle screw placement [7], and clinical outcomes [6].

Many clinical, social, and even economic factors have been extensively analyzed in the literature and shown to influence the length of stay (LOS) after spinal procedures. However, surgeon’s experience was mostly examined relative to a learning curve and not regarding the time in practice when performing minimally invasive transforaminal lumbar interbody fusion (TLIF) [8,9] or anterior cervical discectomy and fusion [5]. Even when comparing hospitalization times for surgeons with more years in practice to their younger colleagues performing complex scoliosis correction surgeries, any potential changes may have been related to the learning curve [6] or different techniques used over a longer period of time [10]. These studies did not find any significant correlations between LOS and surgeon’s experience. On the contrary, the majority of studies examining such surgical procedures as total joint replacement [11,12], pancreaticoduodenectomy [13], thyroidectomy [2], or prostatectomy [14] found direct associations between the LOS, years of surgical practice, and volume of cases.

The primary objective of this study was to determine the effect of one surgeon’s experience on the LOS in patients undergoing one- to two-level TLIFs.

## Materials and methods

The study design was a retrospective cohort study of hospital discharge data. The cohort was comprised of 240 consecutive patients who had undergone open one- or two-level elective TLIF procedures performed for lumbar degenerative disc disease. Patients were selected for surgical treatment based on clinical symptoms and either had spondylolisthesis with or without instability or spinal stenosis requiring extensive decompression. The surgeries were performed at a community-based hospital from March 2011 to April 2018 by a single spine fellowship-trained and board-certified neurosurgeon.

The primary predictor was the surgeon’s experience based upon the years of practice. The primary outcome was LOS, which was controlled by the discharge criteria that remained consistent throughout the study: an afebrile patient tolerating a regular diet, adequate pain control achieved with oral analgesics, ability to self-care, and no evidence of impending complications. Secondary outcomes included surgical time and estimated blood loss (EBL).

Statistical methods

Regression analyses were conducted that included a generalized linear model (GLM) for the Poisson distribution of the LOS and multiple linear regressions for the OR time and EBL against independent factors using R Studio (RStudio Inc., Boston, MA).

Analysis of the patient’s LOS against a surgeon’s experience was conducted via a GLM instead of a linear regression as the response variable is a count variable and would violate the conditions of a linear regression analysis with normality assumption [15]. We used the GLM model for the Poisson distribution with a logit link function. To prove GLM for the Poisson regression was the right model for the data set, a goodness of fit test was performed yielding a p-value of 0.9999 verifying GLM as the ideal test. With the Poisson regression, it is assumed that the mean of the distribution equals the variance [16]. However, this assumption holds true for an ideal data set. Thus, robust standard errors or parameter estimates needed to be calculated to account for mild violation of the Poisson distribution assumption (mean (LOS) = 4.104167 and var (LOS) = 6.269439) in this data set [17].

The strength of the independent factors was used as an estimate to determine if the surgeon’s experience affected the outcomes of interest. The independent factors included patient demographics, admission type (inpatient vs. planned admission from home), intraoperative and immediate postoperative complications stratified using the Clavien-Dindo classification of surgical complications [18], discharge disposition, surgical and clinical parameters, including American Society of Anesthesiologists (ASA) physical status classification score [19]. Based on patient medical history, ASA scores were defined as follows: I, normal healthy patient, II, mild systemic disease, III, severe systemic disease, IV, severe systemic disease that is a constant threat to life, V, a moribund patient who is not expected to survive without the operation. The complications were rated according to the Clavien-Dindo classification grades: 1, any deviation from the normal postoperative course without the need for pharmacological treatment, or surgical, endoscopic, and radiological interventions; 2, requiring pharmacological treatment with drugs other than antiemetics, antipyretics, analgesics, diuretics, and electrolytes; 3, requiring surgical, endoscopic, or radiological intervention; 4, life-threatening complication requiring intermediate care or intensive care unit management; 5, death of a patient.

## Results

There were a total of 240 patients of which 126 patients were female and 114 patients were male. The mean age was 61.9 years (range, 27-88). Further baseline clinical parameters, comorbidities, and surgical characteristics are described in Table 1.

**Table 1:** Selected Patient Demographics, Clinical Characteristics, and Surgical Parameters. ASA, American Society of Anesthesiologists; CD, Clavien-Dindo; EBL, estimated blood loss; LOS, length of stay; OR, operating room; TLIF, transforaminal lumbar interbody fusion

Characteristics	Values
Demographics	
N	240
Age (years)	61.9 ± 13.0
Female (%)	126 (52.5%)
Admission type/inpatients (%)	23 (9.6%)
Clinical parameters	
Previous fusion	51 (21.3%)
Previous non-fusion surgeries	34 (14.2%)
ASA score	I – 16 (6.7%) II – 109 (45.4%) III – 108 (45.0%) IV – 6 (2.5%)
Surgical parameters	
One-level TLIF	139 (57.9%)
Two-level TLIF	101 (42.1%)
TLIF w/additional decompression and posterior lumbar fusion	40 (16.7%)
EBL (mL)	186.0 ± 170.7
Surgical time (minutes)	239.3 ± 59.8
LOS (days)	4.1 ± 2.5
Complications	
CD 1	59 (24.6%)
CD 2	24 (10.0%)
CD 3	8 (3.3%)
CD 5	1 (0.4%)
Discharge disposition	
Home or self-care	135 (56.3%)
Home health organization	48 (20.0%)
Inpatient rehabilitation facility	19 (7.9%)
Skilled nursing facility	38 (15.8%)

LOS against surgeon experience

**Figure 1:**
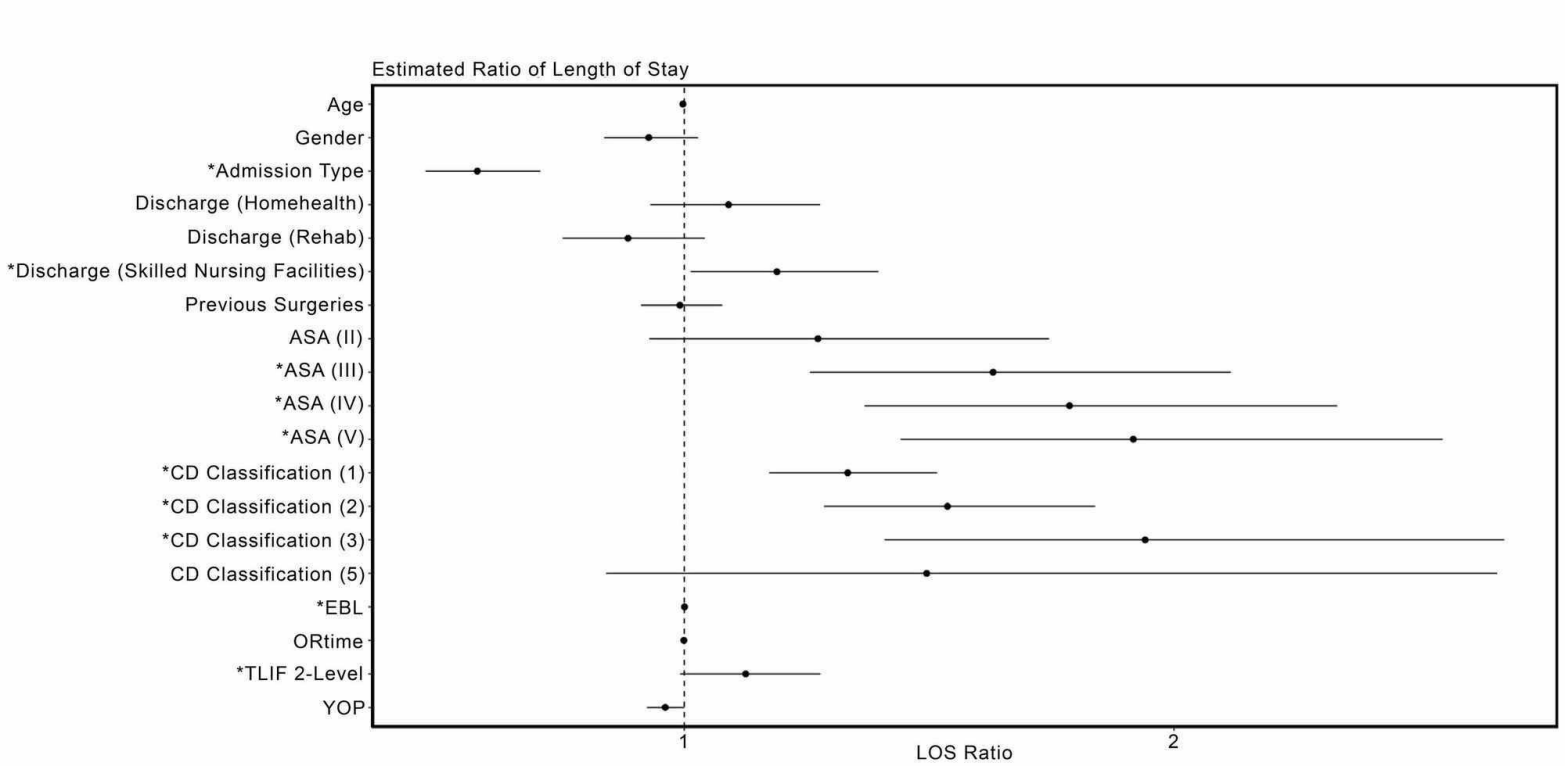
The figure shows the ratios of the variables occurring compared to the length of stay. *Indicates statistically significant variables. ASA, American Society of Anesthesiologists physical status classification score; CD, Clavien-Dindo classification of surgical complications; EBL, estimated blood loss; TLIF, transforaminal lumbar interbody fusion; YOP, years of practice.

Based on the Poisson regression model, it can be inferred that the LOS is not significantly associated with a surgeon’s experience (Pr(>|t|) = 0.8985, CI: -0.5825 to 0.5114) while controlling for all other variables (Figure 1).

Other independent factors did seem to significantly influence patients’ LOS, including the admission type (Pr(>|t|) = 9.637^-08^, CI: -0.8186 to -0.3786). The patients who were admitted from home were discharged significantly quicker than the patients who were undergoing surgery as inpatients. Similarly, the number of TLIF levels (Pr(>|t|) = 1.721^-06^, CI: 0.0606 to 0.1446), the Clavien-Dindo (Pr(>|t|) = 0, CI: 0.1489 to 0.1494), the ASA physical status classification scores (Pr(>|t|) = 4.878^-3^, CI: 0.0336 to 0.1880), being discharged to skilled nursing facility (Pr(>|t|) = 3.44^-2^, CI: 0.0127 to 0.3339), and EBL (Pr(>|t|) = 4.884^-08^, CI: 0.0004 to 0.0008) were found to independently predict LOS. 

Linear regressions were performed in order to analyze secondary outcomes, including surgical time and EBL.

EBL against surgeon experience

A log transformation needed to be applied in order to normalize the data and perform a linear regression analysis. The linear regression analysis for EBL shows that a surgeon’s experience is not a significant factor (Pr(>|t|) = 0.9997, CI: -0.0549 to 0.0595) while controlling for all other variables. As expected, longer surgeries seemed to be significantly associated with an increase in EBL (Pr(>|t|) = 0.0005, CI: 0.0015 to 0.0051).

Surgical time against surgeon experience

When a linear regression analysis was performed on the surgical time, while controlling for all other variables, the surgeon’s experience was found to be highly significant (Pr(>|t| )= 6.30^-16^, CI: -18.4478 to -11.6606) as well the EBL (Pr(>|t|) = 0.0002, CI: 0.0358 to 0.1112) and the number of TLIF levels (Pr(>|t|) = 1.39e-12, CI: 32.4706 to 55.5189) with surgical time.

## Discussion

The healthcare system in the United States is implementing new reforms and strategies in an attempt to provide more efficient care and address the rising costs in healthcare. Advances in surgical technologies and pain management have shortened recovery times and are shifting patient care to an outpatient setting, but the average LOS still was 4.5 days with an average cost of $10,400 per hospitalization according to the Agency for Healthcare Research and Quality report in 2012 [20]. The existing literature identified multifactorial reasons for prolonged hospitalization after lumbar surgeries, including increased age, female gender, body mass index, number of levels fused, surgical time, comorbidities, steroid use, intraoperative blood transfusion and fluid administration, and complications [21-26]. Besides clinical factors, multiple variables may increase LOS; among them are insurance requirements, social factors (lower-income communities), geographical locations (Northeast region) [20] or payment systems, differences in clinical practices, and even the number of hospital beds per capita [27].

When investigating surgeon’s experience, different definitions are used, but it is usually analyzed in relation to a learning curve for the procedure and measured as the surgical time, EBL, complications, and LOS. The learning curve is considered completed when these parameters achieve a steady state. It is also often analyzed as training and the association of volume and outcome. Although all these variables interact, there is another important factor to address: the time in practice or surgeon’s experience that constitutes not only technical ability and perioperative management competency, but also most likely contains a major component of discretion in clinical decision making acquired with time. Therefore, it is important to analyze surgeon’s experience as a continuous variable and not just compare individual surgeons with different experiences.

The main hypothesis of this study was that with experience, surgeons develop more efficient patient clinical management skills and confidence, which would affect the LOS. The study that analyzed variations in physician’s hospitalization practices demonstrated that younger and less experienced physicians kept their patients in hospital longer than their more experienced colleagues [28]. Our study results clearly demonstrated efficiency, as there was a statistically significant reduction in the surgical time, but we did not identify a clear correlation between LOS and surgeon experience overtime suggesting that other factors are likely contributing to such outcome. In our study, patient comorbidities, the number of surgical levels, complications, discharge to a skilled nursing facility, EBL, and whether a patient was inpatient or admitted from home played a statistically significant role. It is also unclear why such discrepancy exists among the published reports on spine and other surgical procedures.

Discharge criteria and duration of stay after spinal procedures vary significantly and are often explained through patient-centered care. Regardless, most of the surgeons do not employ specific discharge criteria or there is no consensus when such criteria are used [29]. Statistically significant LOS variations (from 1.3 to 4.1 days) were noted among surgical centers spine clinics that participated in the Spine Patient Outcomes Research Trial (SPORT) study and for the patients who were undergoing surgery for lumbar stenosis and degenerative spondylolisthesis [30].

Limitations

The study included only one- and two-level TLIF cases performed by a single surgeon in community hospital settings, and although the surgeries were performed over a 6.5-year period, the surgical technique and discharge criteria remained consistent. This allowed us to minimize confounding factors and have a homogeneous patient sample, but at the same time limited the results generalizability.

We have controlled for various independent factors, but the study was not specifically designed to evaluate a number of hospital-, patient-, and provider-level characteristics.

## Conclusions

Based upon the years in practice, surgeon experience was not associated with length of hospitalization and EBL during surgery in patients undergoing one- and two-level TLIF surgeries. However, while controlling for all other variables, the surgeon’s experience and surgical time had a highly significant correlation. The study results clearly demonstrated efficiency, but we did not identify a clear correlation between LOS and surgeon experience overtime suggesting that other factors are likely contributing to such outcome. The average LOS is a complex measure of healthcare resource use and hospital discharge policy or other variables are likely to have more effect on LOS than individual surgeons’ preferences.
